# How to Enhance Gas Removal from Porous Electrodes?

**DOI:** 10.1038/srep38780

**Published:** 2016-12-23

**Authors:** Thomas Kadyk, David Bruce, Michael Eikerling

**Affiliations:** 1 Simon Fraser University, Department of Chemistry, Burnaby, V5A 1S5, Canada; 2 ZincNyx Energy Solutions Inc., Vancouver, V6P 6T3, Canada

## Abstract

This article presents a structure-based modeling approach to optimize gas evolution at an electrolyte-flooded porous electrode. By providing hydrophobic islands as preferential nucleation sites on the surface of the electrode, it is possible to nucleate and grow bubbles outside of the pore space, facilitating their release into the electrolyte. Bubbles that grow at preferential nucleation sites act as a sink for dissolved gas produced in electrode reactions, effectively suctioning it from the electrolyte-filled pores. According to the model, high oversaturation is necessary to nucleate bubbles inside of the pores. The high oversaturation allows establishing large concentration gradients in the pores that drive a diffusion flux towards the preferential nucleation sites. This diffusion flux keeps the pores bubble-free, avoiding deactivation of the electrochemically active surface area of the electrode as well as mechanical stress that would otherwise lead to catalyst degradation. The transport regime of the dissolved gas, viz. diffusion control vs. transfer control at the liquid-gas interface, determines the bubble growth law.

Gas evolution is a vital process in many electrochemical systems. Bubbles appear as the result of primary electrode reactions in electrolysis, e.g., in chlor-alkali or water electrolysis, in the Hall-Hérault process for aluminum production^1^, and in direct-alcohol fuel cells^2^,^3^. They also occur in side reactions, e.g., in the charging of lead acid batteries or in electroplating and electrowinning.

In gas-evolving reactions, the electrode fulfills a twofold function: The electrochemical function of the electrode is to produce dissolved gas. The physical function is to liberate the dissolved product from the liquid by formation of a gaseous phase; in this respect, gas-evolving electrodes fulfill a function similar to other solid interfaces that evolve gas as a result of supersaturation, e.g., due to a decrease of pressure (e.g. cavitation) or increase of temperature (e.g. boiling^4^). This physical process of gas evolution can be divided into four stages: nucleation, growth, detachment and transport of bubbles.

At gas-evolving electrodes, electrochemical and physical processes occur concurrently and they are coupled in two ways: by mass transport and by re-distribution of current density. Bubble-induced mass transport effects exist on both the macro- and the micro-scale. On the macro-scale, the bubbles rise in the electrolyte due to their buoyancy, creating free convective flow^5^,^6^,^7^,^8^, e.g., along vertical electrodes^9^. On the micro-scale, during bubble growth on the surface, liquid is pushed off in radial direction, leading to microconvection^10^. After bubble break-off from the electrode, the volume previously occupied by the bubble is filled, leading to microconvection by wake flow^10^. Bubble growth in micro- and nanoconfinement, i.e., inside of pores, can lead to high mechanical stress in the catalyst structure due to the high capillary pressure. This can contribute to mechanical degradation of catalyst structures^11^,^12^.

The coupling by re-distribution of current density includes two effects: First, the gas fraction in the electrolyte decreases the conductivity of the electrolyte, which can lead to a macroscopic re-distribution of the current density. The second effect, which is the focus of this work, is the blockage and inactivation of part of the active surface area by the adhering bubbles. When part of the surface area is inactivated by bubbles, the remaining uncovered surface has to produce a higher current density to make up for the loss of active area. This drives the overpotential and kinetic losses up.

In classical modeling approaches for flat electrodes, the bubble coverage is often used as an empirical descriptor of this performance loss^13^,^14^,^15^, which is sufficient for engineering purposes. In porous flow-through electrodes, the gas void fraction can be used in a similar fashion to describe how much of the pore volume is filled with gas^16^,^17^. However, despite these rudimentary modeling efforts, the correlation between structural design parameters of porous electrodes like porosity, particle and pore sizes, wettability, catalytic activity on the one hand and overall performance on the other hand remains largely empirical.

Generally, heterogeneous wetting properties have a strong impact on bubble formation and transport. For example, on flat electrodes it was found that by providing hydrophobic islands the rate, size and place of bubble formation can be controlled^18^. With this it is possible to minimize the “foot” of the bubble, i.e., decrease the bubble coverage and maximize performance. This suggests that in porous electrodes it should similarly be possible to control bubble formation and optimize gas transport by tuning the composition and structural design parameters of porous electrodes.

A first step in this endeavour will be taken in this paper. First, a model for bubble growth based on energy considerations is presented. From detailed analyses of the model, the central idea of this paper is derived: controlling bubble formation by introducing artificial preferential nucleation sites. The feasibility of this concept is investigated by coupling the physical model of bubble growth with an electrochemical porous electrode model. Finally, the capabilities of the model are explored with a parameter study and different transport regimes of the dissolved gas are analyzed and discussed.

## Model Development

To tackle the problem of bubble formation at porous electrodes, in this section we start by considering a single bubble that is placed into an electrolyte. Based on simple energy considerations, we develop the bubble growth law that is central to this work. Thereafter, we employ this growth law in a minimalistic electrolyzer model to gain understanding of bubble formation. These insights lead to the idea of preferential nucleation sites. In order to evaluate the feasibility of this concept, the bubble growth law is coupled to a porous electrode model. The Remarks section discusses the limitations of this approach.

### Single Bubble in Electrolyte

As a first step, we consider a single bubble placed freely into aqueous electrolyte without gas transfer across the gas-liquid interface, i.e., no gas dissolution or transfer of dissolved gas into the bubble. If the bubble is in mechanical equilibrium, the surface tension, *γ*, is constant at the bubble surface giving rise to a pressure difference across the liquid-gas interface,


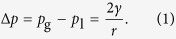


With the ideal gas law, *pV* = *n*R*T*, and a spherical volume of the bubble, *V* = (4/3)*πr*^3^, Equation 1 becomes


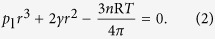


Solving this cubic equation, using Cardano’s method, yields the real root


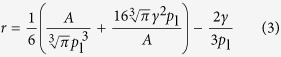


with


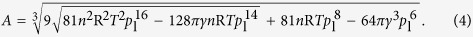


Note that for small bubbles with 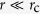, where *r*_c_ = 2*γ*/*p*_1_, this equation simplifies to *r* = (3*n*R*T*/8*πγ*)^1/2^, i.e., 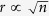, as pointed out by Ljunggren and Erikson^19^. For water and atmospheric pressure, *r*_c_ = 1440 nm.

**Figure 1:**
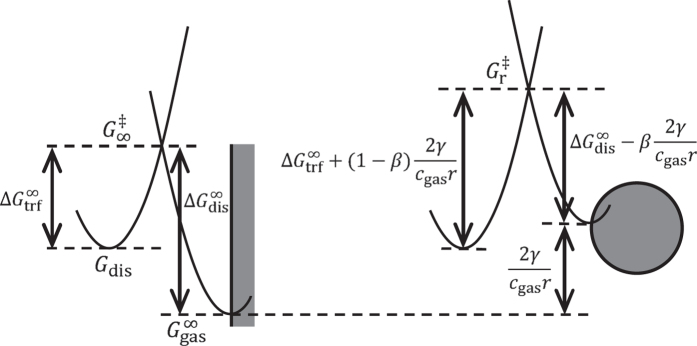
Size dependence of the activation energies of dissolution and transfer processes (schematic). Left: chemical potentials of dissolved and gas phase oxygen at a flat gas-liquid interface. Right: chemical potentials at the spherical gas-liquid interface of a bubble. Adapted with permission from ref. 20. Copyright 2016 American Chemical Society.

Now, let us consider the transfer of gas across the liquid-gas interface using transition state theory. The molar Gibbs energy of oxygen, both dissolved in the electrolyte and in the gas phase, is depicted in Fig. 1. The reaction path for the transfer of gas between the electrolyte and the gas phase passes through a transition state, *G*^‡^. This results in the activation energies for dissolution and transfer, 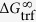 and 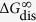. Compared to a flat interface (left figure), the Gibbs energy of the gas in the bubble is increased by the surface energy contribution, 2*γ*/(*c*_gas_*r*). The activation energies are shifted proportionally. Thus, the activation energies become a function of the bubble radius,


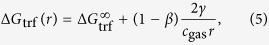



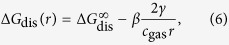


where *β* is the transfer coefficient. Note that similar considerations can be made for solid particles^20^ or liquid droplets. However, since gas bubbles are compressible and the compression depends on the bubble size (Equation 1), the concentration *c*_gas_ = *n*_gas_*V* is a function of bubble radius. The rates for gas transfer into and out of the bubble are


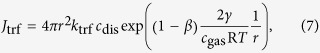



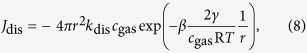


where *c*_dis_ and *c*_gas_ are the concentrations of the dissolved gas in the electrolyte and of the gas in the bubble, respectively. The total flux is


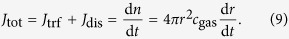


Combining Equations 7, 8 and 9 yields the bubble growth rate


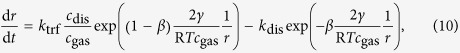


where the concentration of the gas in the bubble can be obtained from the mechanical equilibrium, Equation 1, as


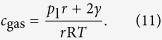


With this, the bubble growth rate becomes


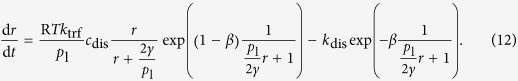


In Equation 12, the right hand side contains terms for the transfer of gas out of and into the bubble. In the limit *r* → 0, the first term vanishes and Equation 12 becomes


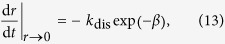


i.e., in small bubbles, the gas transfer out of the bubble dominates and the bubble will dissolve. On the other hand, in the limit *r* → ∞, Equation 12 simplifies to


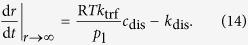


In this case, provided there is sufficient gas dissolved in the electrolyte, the bubble will grow. The critical radius *r*_crit_, marking the transition between dissolution and growth regimes, is found from the condition d*r*/d*t* = 0. Assuming that 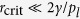 gives


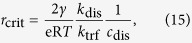


where e is Euler’s number.

Equation 12 is the main equation for the further model development. Therefore, we will illustrate it in a simple thought experiment in the following section, which will lead to the key idea of preferential nucleation sites.

### Minimalistic Electrolyzer Model

Let us consider an electrolyte volume *V*, in which dissolved gas is constantly produced, as it is the case in an electrolyzer under galvanostatic operation. For simplicity, let the dissolved gas be uniformly distributed, which is fulfilled when diffusion is fast compared to gas transfer (ideal mixing limit). In this limit, the dissolved gas can be described with


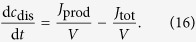


The produced gas flux *J*_prod_ either accumulates as dissolved gas in the electrolyte (left hand side) or it transfers into the gas bubble (flux *J*_tot_). In a galvanostatic electrolyzer, the flux of produced gas is given by the current density *j* as *J*_prod_ = *j*/(*z*_e_F). With the transfer flux from Equation 9, together with Equation 11, we obtain


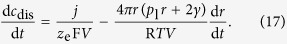


Equations 17 and 12, together with their respective initial conditions, describe our minimal galvanostatic electrolyzer. While the initial condition for the concentration is obvious, *c*_dis_(*t* = 0) = 0, the initial condition for the radius needs more detailed consideration. If we would use *r*(*t* = 0) = 0, then following Equation 13, the radius would decrease un-physically to negative values. Thus, we need a physically meaningful lower boundary for the radius. Brownian motion of gas molecules leads to their collision, triggering the spontaneous formation of gas clusters. These clusters will break up again if they are too small. However, if their size exceeds a nucleation radius *r*_nuc_ they will act as nuclei for bubble formation. These processes can be described in detail with nucleation theory^21^,^22^,^23^; for simplicity, we can use an estimate for *r*_nuc_, say ten times the van der Waals radius of a gas molecule. This estimated value of *r*_nuc_ can then be used as a lower bound for the bubble radius to be integrated in Equation 12.

**Figure 2:**
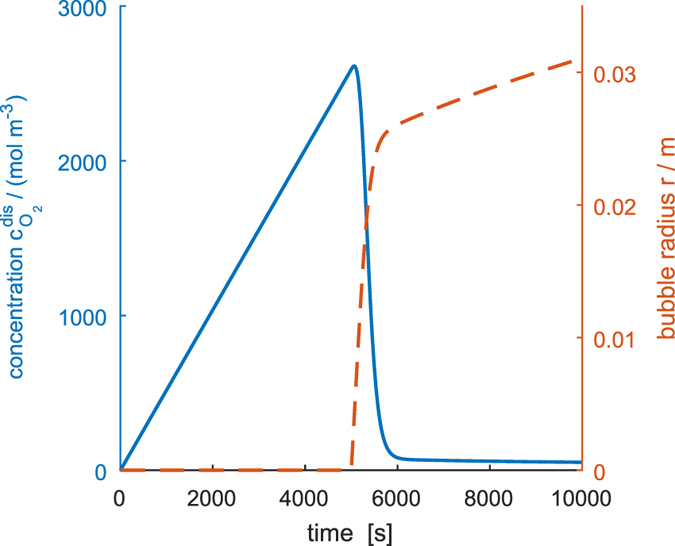
Nucleation and growth of a single bubble under constant current. Solid line: concentration of dissolved gas, dashed line: radius of the bubble.

Simulation results of this simple thought experiment, evaluated for oxygen evolution, can be seen in Fig. 2: after switching the electrolyzer on, it will constantly produce dissolved oxygen. The concentration *c*_dis_ will continuously increase until it attains a critical concentration *c*_nuc_, which is high enough to sustain the growth of the bubble nuclei. Note that this critical concentration is in the order of several hundred times of the saturation concentration. That such high supersaturation is necessary to promote bubble formation has recently been found in experiments on recessed Pt nanopores^24^ for both hydrogen and oxygen evolution.

After a nucleus is transformed into a stable bubble at *c*_dis_ > *c*_nuc_, it grows rapidly while it absorbs the excess dissolved oxygen. This causes a sharp decrease in *c*_dis_, as can be seen in Fig. 2, to a value close to the saturation concentration *c*_sat_. After its initial fast growth to *r* > *r*_nuc_, the bubble continues to grow at a low rate while the concentration remains nearly constant around the saturation concentration. What we can learn from this is that when a bubble is present, it acts as a sink for the dissolved gas and lowers its concentration to values in the order of the saturation concentration. However, if there is no bubble present, much higher concentrations can be reached, before a bubble nucleates. The question is: How can we utilize this effect?

### Preferential Nucleation Sites

**Figure 3:**
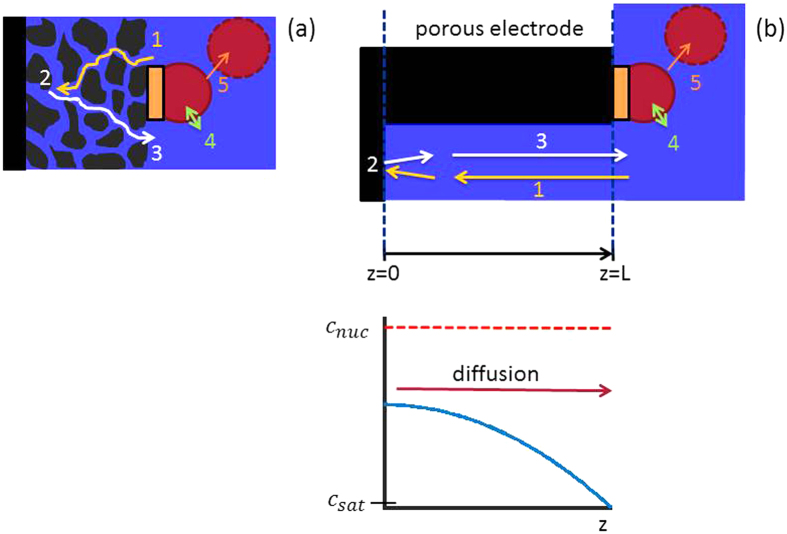
Scheme of (**a**) porous electrode with artificial nucleation sites and (**b**) representation of the electrode as an effective medium with a solid, electron conducting phase (black) and ion conducting electrolyte phase (blue) and corresponding concentration profile of dissolved gas (bottom). Depicted processes are 1 ion transport; 2 electrochemical reaction on the catalyst surface; 3 diffusion of dissolved gas; 4 transfer across the liquid-electrolyte interface; 5 bubble detachment.

Figure 3 shows schematically how we can take advantage of the behavior discussed above. The main idea is to provide artificial preferential nucleation sites on the surface of the porous electrode. This can be done for example by depositing hydrophobic islands (as studied by Brussieux *et al*. on flat electrodes^18^) but other methods of locally changing the surface wettability (e.g. local oxidation or doping) or providing sites at which gas nucleates more easily (e.g. kinks or crevices in the surface) are thinkable. These preferential nucleation sites let the bubbles form where they are most easily removed into the bulk electrolyte and where they do not inflict mechanical stress onto the catalyst structure. Controlling the size of the nucleation sites allows to control the bubble size at detachment and thus the bubble detachment rate, which allows the optimization of bubble removal. While the bubble grows at the preferential nucleation site, it removes the dissolved gas from the solution and keeps the concentration in the vicinity of the bubble close to the saturation concentration, as we discussed in our thought experiment above. The bubble acts as a sink for the dissolved gas and can prevent the formation of gas bubbles in the pores: as long as *c* < *c*_nuc_ no bubbles will form. Since this critical supersaturation is very high, it is possible to establish very high concentration gradients in the pores, which can remove the produced gas by diffusion, as indicated in the bottom Fig. 3.

The condition *c* < *c*_nuc_ will be analyzed in typical porous electrodes in the following sections. What is the value of *c*_nuc_? We can obtain it from our thought experiment (Equations 12 and 17) in two steps: First, we consider a flat liquid-gas interface, i.e., *r* → ∞, in equilibrium, i.e., d*c*/d*t* = 0 and 
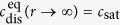
. Thus, Equation 12 becomes Equation 14 and inserted into Equation 17 it gives


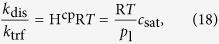


which reproduces Henry’s law with Henry’s constant H^cp^ = 1/(R*T* )· *k*_dis_/*k*_trf_. Inserted into Equation 15, the following relation is found


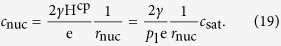


Inserting the estimate for the nucleation radius *r*_nuc_ = 1.5 nm gives *c*_nuc_ = 350 · *c*_sat_, which is in the same order as the experimentally found value^24^. Reversely, the experimentally measured *c*_nuc_ = 0.25 M can be used in Equation 19 to estimate *r*_nuc_ = 1.7 nm.

### Porous Electrode Model

In order to evaluate if by providing preferential nucleation sites the pores of a porous electrode can be kept bubble free, we explore the “toy model” in Fig. 3a. The model domain consists of two parts: the porous electrode of thickness *L* and the surface-adjacent region which contains the preferential nucleation sites. The porous electrode is treated as an effective medium that consists of a solid, electron conducting phase and an electrolyte phase as illustrated in Fig. 3b. In the porous electrode, the electrical double layer and the faradaic surface reaction that produces dissolved gas species as well as the transport of ions, electrons and dissolved gas in the though-plane direction are considered. In the surface domain, the nucleation and growth of bubbles are considered. In the following, we discuss the charge balance equations for the electrolyte and solid phase as well as the material balance of the dissolved gas.

#### Charge Balance for Electrolyte

We assume a high ion concentration in the electrolyte. With this assumption, the double layer is very thin and the ion concentration is nearly uniform. The thin double layer is modeled as a Helmholtz capacitance with an effective double layer capacitance *C*_dl_. The ion concentration at the reaction plane is assumed to have the same value as the bulk electrolyte, i.e., desalination effects as modeled, e.g., by Biesheuvel and Bazant^25^ are neglected. Thus, a Frumkin correction of the Butler-Volmer equation as suggested in ref. 26 is obsolete. Under the assumption of electroneutrality, the charge balance in the electrolyte (liquid phase, l) in the pores can be described as


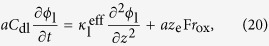


where *ϕ*_1_ is the potential in the electrolyte phase, *a* is the volume-specific active surface area, *z*_e_ is the number of electrons that are exchanged in the reaction and 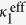 is the effective conductivity of the electrolyte. The term on the left hand side (LHS) describes the charge accumulation in the double layer. The first term on the RHS describes ion migration in the electric field. The second term on the RHS describes the consumption of negative charges (which is equivalent with the production of positive charges, hence the positive sign) in the faradaic reaction with reaction rate *r*_ox_.

The initial and boundary conditions to solve Equation 20 under galvanostatic or potentiostatic operation conditions are


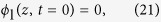



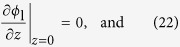



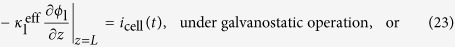



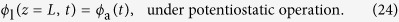


#### Charge Balance for Solid Phase

The potential distribution inside the electron-conducting phase is neglected because of the high effective electronic conductivity compared to the effective ion conductivity. A uniform potential distribution is assumed,


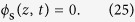


If the electron conductivity is low, e.g., in metal oxide catalysts, a full charge balance of the electrons in the solid phase as outlined in refs 27,28 can be used.

#### Material Balance of Dissolved Gas

The material balance for the gas that is produced in dissolved form is given by


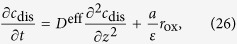


where *ε* is the porosity. The LHS describes the accumulation of dissolved gas in the electrolyte, which is transported by diffusion (RHS, first term) and is produced in the faradaic reaction (RHS, second term). Diffusion is described with the effective diffusivity *D*^eff^, which is assumed to be independent of concentration. Interactions with the pore wall are assumed to be negligible.

The initial and boundary conditions for Equation 26 are


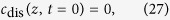



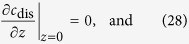



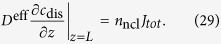


The end of the pore is assumed to be gas-tight. At the mouth of the pore, it is assumed that all gas that exits the pore transfers into the bubble. The diffusion from the mouth of the pore to the surface of the bubble is assumed to be fast, keeping the concentration outside of the pore uniform.

In the second boundary condition, the flux *J*_tot_ couples the porous electrode domain to the surface-adjacent domain via Equation 9. The growth of the bubble, d*r*/d*t*, in the surface-adjacent domain is given by Equation 12, which completes the model. The lower bound as well as the initial condition for the radius is given by *r*_nuc_,


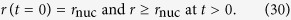


When the bubble has grown to a critical size, it will detach from the nucleation site and the next bubble can grow. The size of the bubble at detachment is determined by a mechanical force balance including buoyancy, pressure, drag, inertia, capillary and lift forces. A detailed model of bubble detachment is beyond the scope of this work. Instead, we use the the size of the bubble at detachment, *r*_det_, as an effective parameter, which could be obtained from experiments or detailed theoretical studies. Our model assumes that upon reaching *r*_det_, the bubble immediately detaches, resetting the bubble radius to the nucleation radius *r*_nuc_.

#### Kinetic Equations

The rate *r*_ox_ of the gas-producing oxidation reaction of the type *ion*^−^ ↔ *gas*(*dis*) + *e*^−^ can be described by the Butler-Volmer equation,


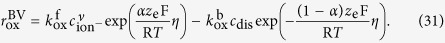


At high overpotential the backward reaction (second term on the RHS) becomes negligible. Furthermore, since we assume the ion concentration to be uniform in the first term on the RHS, the concentration dependence can be neglected and we use a simple Tafel equation,


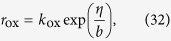


where *k*_ox_ is the surface area-specific oxidation rate constant and *b* is the Tafel slope.

### Remarks

The developed porous electrode model uses effective medium theory to describe the transport of ions and dissolved gas. In the simplest variant, Bruggeman’s equation could be used to determine the effective ion conductivity and the diffusion coefficient,


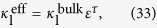



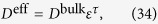


where *τ* is the tortuosity. Since our model assumes uniform conditions for each nucleation site, it represents the porous medium as a periodically repeated unit cell consisting of a single nucleation site, as depicted in Fig. 3. As we will see in the next section, this unit cell shows quasi-periodic behaviour, which results in concentration waves through the porous electrode. For waves through porous media it is known that they are are dampened by both an attenuation due to the limited transport coefficient and scattering dissipation due to the irregular structure of the porous medium^29^. By using the Bruggeman equations above, the attenuation effect is captured in our model, but scattering dissipation is neglected. Incorporating it would require additional statistical descriptors of the structure of the porous electrode (e.g. pore size distribution) and would lead to additional complexity of the model, which is beyond the scope of this work.

A second effect that is neglected in our unit cell model is the interaction between multiple artificial nucleation sites. Each nucleation site can be seen as an oscillator; depending on the coupling between these oscillators, complex spatiotemporal patterns can form^30^,^31^,^32^. If the oscillators are strongly coupled to each other, they oscillate synchronously; our model results in this scenario because of the periodic repetition of the unit cell. If the oscillators are decoupled from each other, a chaotic pattern emerges. In between these extremes, more complex oscillation patterns are possible. In our case, the coupling between the oscillators occurs via diffusion of the dissolved gas as well as mechanisms that are more specific to bubble formation: coalescence of neighbouring bubbles and bubble-induced convection that can lead to detachment of neighbouring bubbles. Since these complex phenomena are not the focus of this paper, in the following section we focus on the results of a single nucleation site on a unit cell of the porous electrode.

A practical way to address these issues would be to use an effective concentration which represents the average concentration over one oscillation of duration *τ*_osc_,


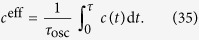


## Results

In this section, we first want to evaluate whether it is feasible to apply preferential nucleation sites in order to keep porous electrodes free of bubbles. For this purpose, we parametrize the general model for the specific case of alkaline oxygen evolution on a structured Nickel electrode in KOH. For the electrode structure, which is represented by *L* and *a*, we assume an inverse opal structure^33^,^34^,^35^. This structure can be made by templating with polystyrene spheres, electrodeposition of Nickel in the void between the spheres and subsequent removal of the templates. The advantages of this structure are that it is well defined, allows direct control over the structural parameters (radius of the inverse opals, thickness of the layer via number of layers) and can easily be manufactured^33^,^34^,^35^. The parameters used in the following are summarized in Table 1. For the transfer rate constant *k*_trf_ a parameter study was performed, since gas transfer is the most sensitive process in the system.

**Figure 4:**
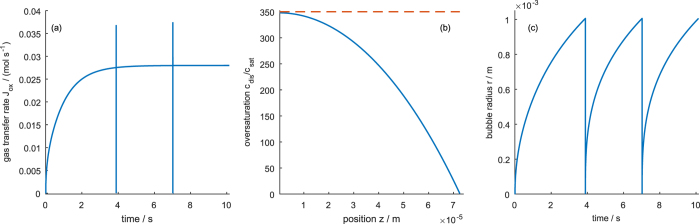
Evolution of (**a**) gas transfer rate *J*_tot_(*t*), (**b**) oversaturation profile *c*_dis_(*z*)/*c*_sat_, and (**c**) bubble radius evolution *r*(*t*) at high gas transfer rate *k*_trf_ = 1 m s^−1^. Dashed line in (**b**) marks the critical oversaturation, *c*_nuc_/*c*_sat_, above which bubble nucleation occurs.

Figure 4 shows the oversaturation of dissolved gas, *c*_dis_/*c*_sat_, the transfer flux, *J*_tot_, and the bubble radius for a high transfer rate constant across the liquid-gas interface. After the electrolyzer is switched on, the concentration of dissolved gas increases until a stable steady state profile is reached. Close to the mouth of the pore, *z* = *L*, the concentration is close to the saturation concentration (Fig. 4b). Towards the end of the pore, *z* = 0, the concentration increases, but the diffusion flux is large enough to keep the concentration well below the critical concentration at which bubbles would nucleate in the pores. Since transfer is fast, diffusion is the limiting process that determines the concentration profile. Due to this diffusion control, the concentration profile is unaffected by the bubble, i.e., the periodic growth of the bubble does not influence the concentration profile. The gas transfer rate in Fig. 4a shows that after an initial increase, a nearly constant gas removal is achieved that is only interrupted by small “ripples” each time a bubble detaches. The evolution of the bubble radius in Fig. 4 shows a square-root like growth of the bubble, similar to experimental results observed on flat electrodes with hydrophobic patches^18^.

**Figure 5:**
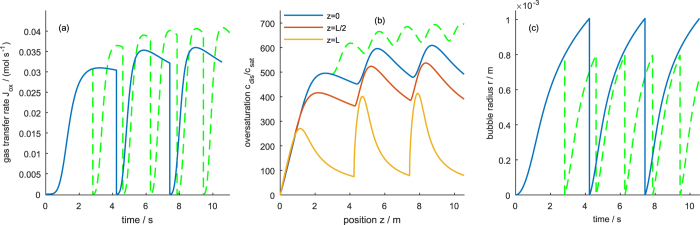
Evolution of (**a**) gas transfer rate *J*_tot_(*t*), (**b**) oversaturation *c*_dis_(*t*)/*c*_sat_ at the bottom (blue), middle (red) and mouth of the pore (yellow); and (**c**) bubble radius *r*(*t*) at intermediate gas transfer rate *k*_trf_ = 10^−5^ m s^−1^. Green dashed lines show the respective evolution when the bubble detachment size is reduced to half the amount of gas.

When the transfer rate constant is reduced to medium values, the “ripples” in the gas transfer rate become more pronounced (*cf*. Fig. 5a) and show an overshoot. As can be seen in Fig. 5b, these ripples send concentration waves from the mouth to the bottom of the pores. Since the bubble detachment occurs periodically, so do the concentration waves and the concentration does not reach a stationary value but oscillates. This behavior is caused by a mixed regime controlled by both diffusion and transfer. In this regime, the bubble growth law changes: while under diffusion control, bubble growth follows a concave curve, in the mixed regime the bubble growth starts convex, goes through an inflexion point and ends concave.

**Figure 6:**
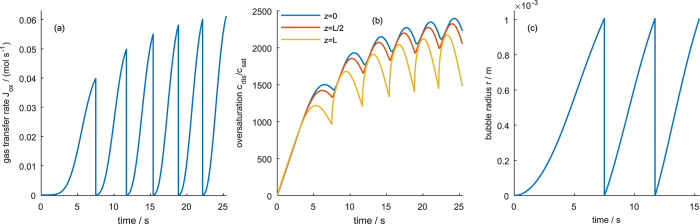
Evolution of (**a**) gas transfer rate *J*_tot_(*t*), (**b**) oversaturation *c*_dis_(*t*)/*c*_sat_ at the bottom (blue), middle (red) and mouth of the pore (yellow); and (**c**) bubble radius *r*(*t*) at low gas transfer rate *k*_trf_ = 10^−6^ m s^−1^.

Lowering *k*_trf_ further to very small values leads to the behavior seen in Fig. 6: the gas transfer rate now oscillates between zero and a local maximum. The enclosing curve approaches an asymptotic limit. At the same time, the bubble frequency increases. The concentration oscillates and follows the trend of the gas transfer rate, i.e., the transport regime is now controlled by the gas transfer into the bubble. Noteworthy is that the concentration now reaches values that are higher than the critical concentration and bubbles would start to form inside the porous electrode. Under transfer controlled conditions, the bubble growth is linear after reaching stationary conditions. This implies that the transport regime determines the bubble growth law (compare Figs 4c, 5c and 6c).

**Figure 7:**
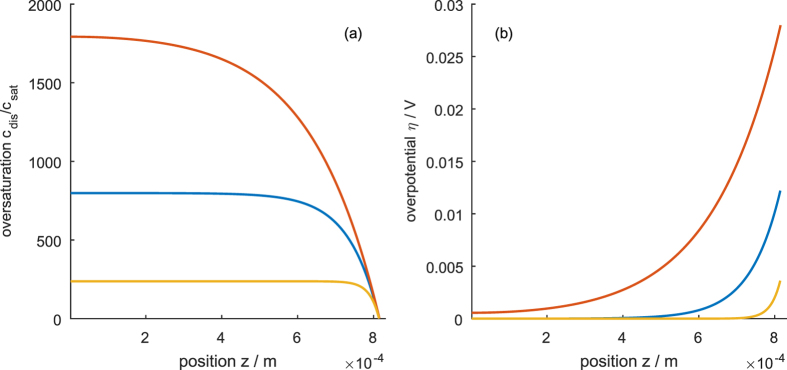
Influence of the specific surface area on the concentration (**a**) and overpotential (**b**) profile at an electrode thickness greater than the reaction penetration depth. Blue is the reference case with a = 4.44 · 10^6^ m^2^ m^−3^, red shows a ten times decreased specific surface area and yellow a ten times increased specific surface area.

**Figure 8:**
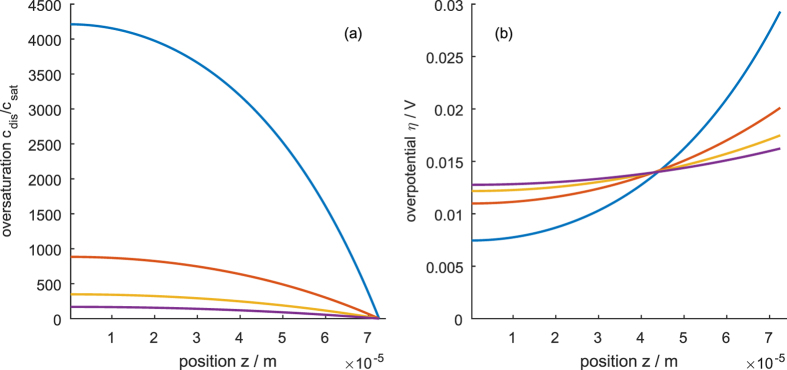
Influence of the porosity on (**a**) concentration profile and (**b**) overpotential profile for an electrode thickness below the reaction penetration depth. Porosity of 25% (blue), 50% (red), 74% (corresponding to an inverse opal structure; yellow), 100% (purple).

**Figure 9:**
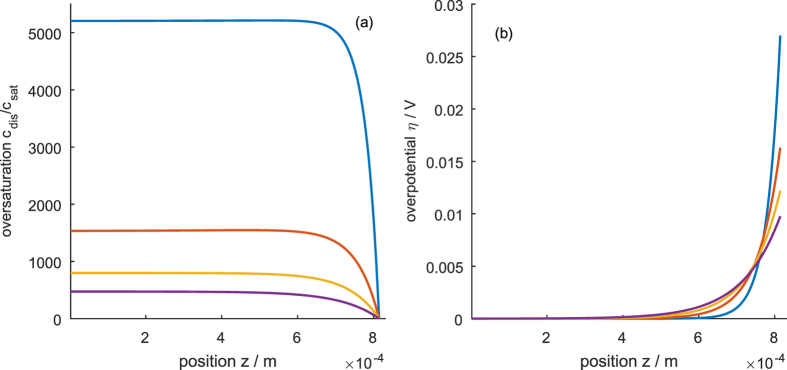
Influence of the porosity on (**a**) concentration profile and (**b**) overpotential profile for an electrode thickness above the reaction penetration depth. Porosity of 25% (blue), 50% (red), 74% (corresponding to an inverse opal structure; yellow), 100% (purple).

Figures 7b, 8b and 9b show the distribution of the overpotential across the thickness of the electrode for operation at 200 mA cm^−2^ for different electrode designs discussed in the next section. Generally, the overpotential and the reaction rate are highest at the mouth of the pores. If the electrode is too thick or the ion conductivity is too low, the ion transport limitation can result in the inactivation of parts of the pores. On the other hand, if the electrode is too thin, the surface enhancement of the porous electrode decreases, thus increasing the overpotential. Thus, an optimum thickness lies in between; its value can be estimated using the concept of the reaction penetration depth *l*_c_^36^,^37^, an intrinsic electrode parameter that describes the competition of the reactant conversion ability and the ion conductivity. At small overpotentials, it is the characteristic length scale of the exponential decay of the local overpotential and reaction rate and is given by


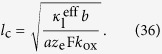


Noteworthy is that the overpotential reaches a steady state profile and no bubble-induced potential oscillations occur. This becomes clear when analyzing the model structure: the kinetic equation for the reaction rate, Equation 32, couples the charge balance, Equation 20, and the material balance, Equation 26, since *r*_ox_ appears in both of them. When operating away from equilibrium at large overpotentials (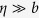), the *c*_dis_-dependent backwards reaction becomes negligible and the concentration dependency of the reaction rate disappears (*cf*. Equation 31 vs. 32). The charge balance influences the material balance by dictating where the gas is produced, but concentration has no influence on potential, i.e., mass transfer by diffusion and transfer into the bubble do not affect the potential.

### Comparison with Experiments on Flat Electrodes

**Figure 10:**
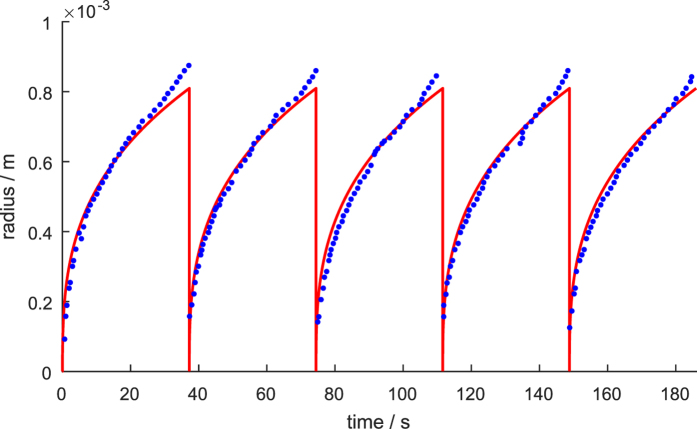
Fitting of experimental data of Brussieux *et al*.^18^ of bubble growth at flat electrodes at 20 mA cm^−2^.

Figure 10 shows a model fit to experimental data from Brussieux *et al*.^18^, who measured bubble growth on hydrophobic islands on flat electrodes. For the fit, only the bubble detachment size *r*_det_ was fitted. The other parameters like current density and active surface area, were adjusted according to the experimental conditions. The model reproduces the bubble growth well, except in the range of large bubbles, at which bubble deformation and necking of the bubble prior to detachment occurs. The high transfer coefficient used in the fit, *k*_trf_ = 1 suggests the bubbles grow in the diffusion-limited regime. Noteworthy is that with a constant bubble detachment size of *r*_det_ = 0.81 mm, the bubble detachment times are well reproduced, while attempting to fit the detachment size of each bubble individually leads to an ill fit of the detachment times (not shown). This indicates that the size of the hydrophobic island controls the effective bubble detachment size.

### Guidelines for Porous Electrode Design

In order to gain insight to the question, how to design the porous electrode with preferential nucleation sites in order to yield optimal performance, a parameter study was performed. The structural parameters *a, L, ε* and the detachment radius of the bubble, which is determined by the size of the artificial nucleation site, where analyzed.

**Figure 11:**
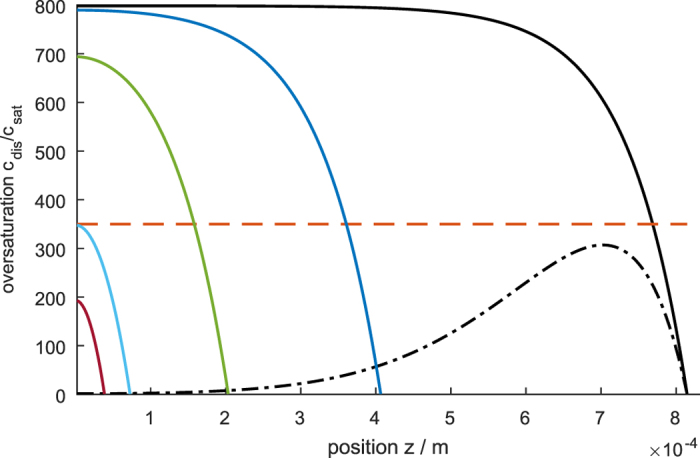
Influence of porous electrode thickness on steady-state concentration profiles. The dashed black line is a transient concentration profile. The dash-dotted red line marks the critical oversaturation for bubble formation, *c*_nuc_/*c*_sat_.

Figure 11 shows the influence of the electrode thickness on the steady-state concentration profile. In the limit of thin electrodes, at which the reaction penetration depth is smaller than the thickness of the electrode, *L* < *l*_c_, the reaction rate and potential are distributed uniformly; an increase in thickness increases the concentration that is reached at the bottom of the pore, *z* = 0. In the thin electrode limit, the thickness should be chosen so the concentration at the bottom of the pore remains below *c*_nuc_. In our example, the limiting case is the light blue curve in Fig. 11. Under typical operating conditions of an electrolyzer, the critical concentration for bubble formation, *c*_nuc_, would be reached before we leave the limit of thin electrodes. This is because the diffusion of the dissolved gas is limiting rather than the electric field driven ion migration that determines the reaction penetration depth.

However, in special applications with low ion concentrations, e.g., in water disinfection electrolysis^38^,^39^,^40^, ion conduction could be limiting in thick electrodes without reaching the critical concentration. In this thick electrode limit with *L* > *l*_c_, increasing the electrode thickness has no effect on the performance; it simply increases the inactive part of the electrode (*cf*. blue and black curves in Fig. 11. During the start-up of the electrolyzer (dashed curve in Fig. 11), gas is produced in the active part of the electrode leading to a concentration profile with a gradient to both the mouth and the bottom of the pore. Thus, the dissolved gas diffuses into the inactive part of the electrode, until a flat concentration profile is reached in this part (solid black line in Fig. 11). The time until this steady state is reached depends linearly on the thickness of the electrode.

The second important structural parameter is the specific surface area *a*. In the thin electrode limit under galvanostatic operation, the production rate of the dissolved gas is constant and uniformly distributed. Hence, the concentration profile and the bubble growth are independent of *a* in this limit. However, *a* directly influences the overpotential according to the Tafel kinetics, Equation 32, i.e., an enhancement of the specific surface area by a factor *ξ* = *a*/*a*^ref^ reduces the overpotential by


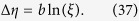


Since *a* changes the reaction penetration depth, *a* ∝ (1/*l*_c_)^2^ (*cf*. Equation 36), in the thick electrode limit, *a* affects the concentration and potential profiles as shown in Fig. 7. Increasing *a* leads to lower overpotential and concentration, but increases the inactive part of the electrode. Hence, when increasing *a* the thickness of the electrode can be reduced accordingly (by a factor of 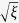).

The influence of porosity in the thin and thick electrode limit is shown in Figs 8 and 9, respectively. In general, the porosity changes both the effective ionic conductivity and the effective diffusion coefficient according to Bruggeman’s equation, Equations 33 and 34. The change in the diffusion coefficient is dominant and strongly influences the concentration in the pore (*cf*. Figs 8a and 9a). The change in ion conductivity leads to an insignificant change in the overpotential in the thin electrode limit (*cf*. Fig. 8b) and to a moderate change of the overpotential in the thick electrode limit (*cf*. Fig. 9b). Generally, *ε* changes the reaction penetration depth with the proportionality *l*_c_ ∝ *ε*^*τ*/2^. The reaction penetration depth can vary from zero for *ε* = 0 to the value of the bulk electrolyte, which is given by Equation 36, for *ε* = 1.

The size of the nucleation site determines the detachment size of the bubble and thus the frequency of bubble detachment, as shown with the dashed line in Fig. 5. The steady-state detachment frequency can be obtained from the consideration that in steady state all the produced gas is removed in the form of bubbles. Thus, the steady state bubble frequency 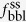 can be obtained under consideration of Equation 2 as


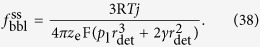


In the range of constant gas transfer as shown in Fig. 4a, i.e., under diffusion limitation, the size of the nucleation site only influences the bubble frequency, but the concentration profile and overpotential remain constant. However, in the transfer controlled or mixed regime, the nucleation site influences the concentration as shown in Fig. 5b: if the nucleation site is made smaller, the bubble spends a larger percentage of the time growing in the transfer controlled regime, which decreases the average bubble growth and gas removal. In turn, the concentration of dissolved gas increases.

## Summary and Conclusions

This work explored the fundamentals of gas removal from porous electrodes. By gaining a basic understanding of the relationships between structure, properties and performance of porous gas-evolving electrodes, their composition and structure can be tuned to control bubble formation and optimize gas transport. A first step in this endeavour was taken by modeling the nucleation and growth of a bubble based on chemical energy considerations. This growth model can explain the experimentally found high oversaturation that is necessary to nucleate bubbles^24^. Additionally, the size of the bubble nucleus in the experiment was estimated.

From detailed analyses of the bubble growth model, the idea of controlling bubble release from porous electrodes by introducing preferential nucleation sites was developed. By providing preferential nucleation sites on the surface of the electrode, e.g., in the form of hydrophobic islands or other means of modifying surface wettability, it is possible to nucleate and grow the bubbles outside of the electrode, where they are most easily removed into the electrolyte and do not exert mechanical stress onto the electrode structure that can lead to degradation or catalyst destruction. While the bubbles grow at the preferential nucleation sites, they act as a sink for dissolved gas, extracting it from the electrolyte and keeping its concentration close to saturation at the surface of the electrode. Because high oversaturation would be necessary to nucleate bubbles inside the pores, large concentration gradients within the porous electrodes can be established, which can drive a diffusion flux towards the preferential nucleation site on the surface, effectively removing the produced gas while keeping the pore space bubble-free.

Based on the bubble growth model, a structure-based model of a gas-evolving electrode was developed and utilized to study a porous electrode with preferential nucleation sites on the surface. For typical porous electrode structures it was found that the concept is feasible. The model also revealed that different transport regimes, viz. diffusion controlled vs. transport controlled by the gas transfer through the liquid-gas interface, would lead to different bubble growth laws: diffusion controlled growth leads to approximately square-root growth while transfer control would lead to approximately linear growth of the bubble radius over time. Evaluation of experiments suggest diffusion control, an assumption that is often made in classical bubble growth theories^19^,^41^.

Based on a parameter study of the structural parameters, guidelines for the design of the porous electrode with preferential nucleation sites were developed. Generally, the aim is to keep the concentration in the pores below the critical concentration for bubble nucleation, while on the other hand maximizing the electrochemically active surface area. In typical electrolytes, gas diffusion is the determining process and dictates the maximum thickness of the porous electrodes. In this limit, the reaction penetration depth is larger than the thickness, and the overpotential as well as the reaction rate are uniformly distributed. An enhancement of the internal surface area leads to a logarithmic decrease of the overpotential. The size of the artificial nucleation site determines the detachment size of the bubble and the frequency of bubble detachment.

## Additional Information

**How to cite this article**: Kadyk, T. *et al*. How to Enhance Gas Removal from Porous Electrodes? *Sci. Rep.* **6**, 38780; doi: 10.1038/srep38780 (2016).

**Publisher's note:** Springer Nature remains neutral with regard to jurisdictional claims in published maps and institutional affiliations.

## Figures and Tables

**Table 1:** Model parameters for alkaline oxygen evolution on inverse opal Nickel electrodes.

Parameter	Value
L	20 *μ*m (25 closed-packed layers of d = 1 *μ*m spheres)
a	4.44 · 10^6^ m^2^ m^−3^
*ε*	0.74
*C*_dl_	0.2 F 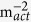
*k*_ox_	1.23 · 10^−4^ mol 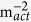 s^−1^
*b*	62.1 mV
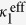	12.74 S m^−1^
*D*^eff^	2.1 · 10^−9^ m^−2^ s^−1^
*β*	0.5
*γ*	0.0728 N m^−1^
*r*_det_	1 mm
*p*_1_	101325 Pa
*T*	298 K
*i*_cell_	200 mA cm^−2^
